# Exploiting β-Cyclodextrin in Molecular Imprinting for Achieving Recognition of Benzylparaben in Aqueous Media

**DOI:** 10.3390/ijms16023656

**Published:** 2015-02-06

**Authors:** Saliza Asman, Sharifah Mohamad, Norazilawati Muhamad Sarih

**Affiliations:** 1 Department of Chemistry, Faculty of Science, University of Malaya, Lembah Pantai 50603, Kuala Lumpur, Malaysia; E-Mails: saliza_asman@yahoo.com (S.A.); nmsarih@um.edu.my (N.M.S.); 2 Department of Science and Mathematics, Faculty of Science, Technology and Human Development, University of Tun Hussein Onn Malaysia, Parit Raja 86400, Johor, Malaysia

**Keywords:** molecularly imprinted polymer, methacrylic acid functionalized β-cyclodextrin, benzylparaben

## Abstract

The molecularly imprinted polymer (MIP) based on methacrylic acid functionalized β-cyclodextrin (MAA-β-CD) monomer was synthesized for the purpose of selective recognition of benzylparaben (BzP). The MAA-β-CD monomer was produced by bridging a methacrylic acid (MAA) and β-cyclodextrin (β-CD) using toluene-2,4-diisocyanate (TDI) by reacting the –OH group of MAA and one of the primary –OH groups of β-CD. This monomer comprised of triple interactions that included an inclusion complex, π–π interaction, and hydrogen bonding. To demonstrate β-CD performance in MIPs, two MIPs were prepared; molecularly imprinted polymer-methacrylic acid functionalized β-cyclodextrin, MIP(MAA-β-CD), and molecularly imprinted polymer-methacrylic acid, MIP(MAA); both prepared by a reversible addition fragmentation chain transfer polymerization (RAFT) in the bulk polymerization process. Both MIPs were characterized using the Fourier Transform Infrared Spectroscopy (FTIR), Field Emission Scanning Electron Microscopy (FESEM), and Brunauer-Emmett-Teller (BET). The presence of β-CD not only influenced the morphological structure, it also affected the specific surface area, average pore diameter, and total pore volume of the MIP. The rebinding of the imprinting effect was evaluated in binding experiments, which proved that the β-CD contributed significantly to the enhancement of the recognition affinity and selective adsorption of the MIP.

## 1. Introduction

Molecular imprinting of synthetic polymers is an advance technique, where monomers and cross-linkers are copolymerized in the presence of a template molecule. The removal of the template molecule from the obtained polymer by simple solvent extraction reveals the complementary binding sites that recognize the template molecule from its structurally similar compounds [1]. Due to their high mechanical and chemical stabilities, ease of preparation, and suitability for a wide range of operating conditions, molecularly imprinted polymers (MIPs) have been developed in various fields, such as solid phase extraction [2], chromatographic separation [3], catalysis [4], membranes [5], and sensors [6].

However, conventional MIPs have low capacity and poor site accessibility for template molecules. In order to overcome this limitation, an appropriate modification of the monomer with β-cyclodextrin (β-CD) has been made to improve the binding capacity of the MIP [7]. β-cyclodextrin (β-CD) is a cyclic oligosaccharide, consisting of seven glucose unit residue linked with α-(1,4) bonds, which has the primary hydroxyl groups on the narrow (primary) side, and the secondary hydroxyl on the inner cavity, as well as a hydrophilic external surface. Due to its unique structure of a truncated cone-shaped molecule, it ideally forms an inclusion compound with various analytes by “host-guest interaction” [8]. Hence, the orientation of the β-CD molecule residues in the MIPs is suitable for the cooperative binding of the templates [9]. The preparation of β-CD-MIPs has previously been reported to be successful [10,11,12,13]. The modification of the monomer with β-CD is a promising step, as there is a lack of specific binding sites in the cavities created by imprinting. By linking several functional groups of monomer to β-CD, the recognition ability could be improved, which increases the binding capacity of MIPs [14].

**Figure 1:**
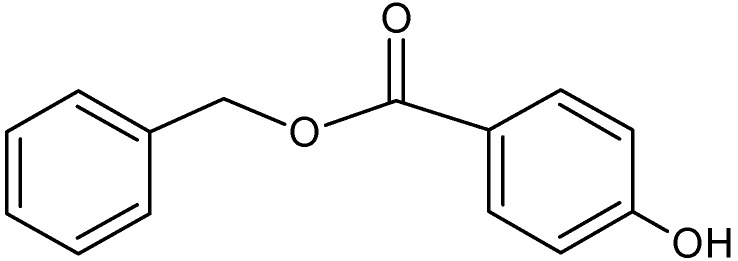
Benzylparaben (BzP).

Benzylparaben (BzP) (Figure 1) is one of the homologous series of parabens, including methyl-, ethyl-, propyl-, butyl-, and benzylparabens. BzP differs slightly from alkylparabens, due to the presence of benzene rings instead of the alkyl chains. Parabens molecules possess an antimicrobial activity, which is widely used in cosmetic and pharmaceutical products [15,16]. However, a possible relationship between breast cancer and prolonged dermal expositions to products containing parabens has been reported [17,18,19,20]. The toxic materials of widely-used parabens are continuously released into aquatic media via domestic wastewater [21]. Parabens have been classified as an emerging environmental pollutant by the U.S. Environmental Protection Agency (USEPA) and the EU’s EMEA (European Medicines Agency) Environment Risk Assessment.

In this report, we describe a detailed study of the influence of β-CD on the characteristics of MIPs. The utilization of methacrylic acid functionalized β-cyclodextrin (MAA-β-CD) as a monomer in molecular imprinting represented molecularly imprinted polymer-methacrylic acid functionalized β-cyclodextrin, MIP (MAA-β-CD) was investigated. In order to gain further insights on the potential benefits in recognizing the properties of MIPs, the molecularly imprinted polymer-methacrylic acid, MIP (MAA) without β-CD was synthesized. Both MIPs were prepared by the reversible addition fragmentation chain transfer (RAFT) polymerization in the bulk polymerization process. RAFT polymerization was selected since it is versatile system and a promising method [22,23]. BzP analyte was selected as the targeted template because our previous study [24], indicated that BzP demonstrated the highest removal using β-CD cross-linked polymer compared to other parabens. The MIPs were characterized using Fourier Transform Infrared Spectroscopy (FTIR), Field Emission Scanning Electron Microscopy (FESEM), and Brunauer-Emmett-Teller (BET). The recognition ability and the binding specificity of the MIPs were also studied, and the interaction of the MAA-β-CD with BzP was studied. Thus, the effect of MIP characteristic on the adsorption and selectivity performances was explored. 

## 2. Results and Discussion

### 2.1. Characterization of MAA-β-CD Monomer

**Figure 2:**
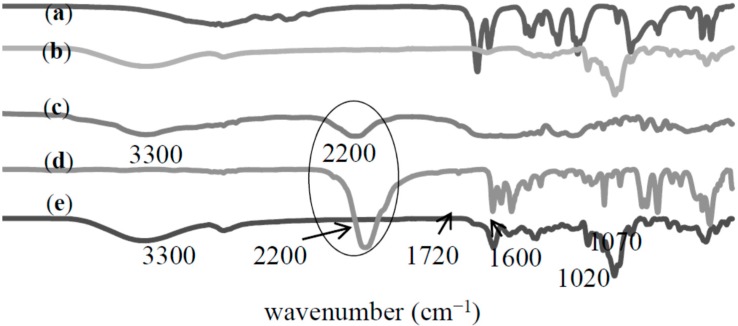
FTIR spectra of (a) methacrylic acid (MAA); (b) β-cyclodextrin (β-CD); (c) methacrylic acid bonded toluene-2,4-diisocyanate (MAA-TDI); (d) toluene-2,4-diisocyanate (TDI); (e) methacrylic acid functionalized β-cyclodextrin (MAA-β-CD).

The formation of MAA-β-CD monomer was characterized using FTIR analysis. Figure 2 shows the spectra of (a) MAA, (b) β-CD, (c) MAA-TDI, (d) TDI, and (e) MAA-β-CD. The spectrum of MAA-TDI shows a peak at 3300 cm^−1^ corresponding to the carbamate group (–NHCO). The presence of a peak at 2200 cm^−1^, which is characteristic of the –NCO group of TDI showed the availability of un-reacted ortho –NCO groups. It is well known that TDI has two functional NCO groups with one in ortho (2) and the other in para (4) positions. The NCO group in the para position is far more reactive than the one in the ortho position [25]. The MAA preferentially reacts at the para position; therefore, in the case of stoichiometric ratio between MAA and TDI, the reaction was considered to always occur at the para position when a catalyst is present [26]. The retained ortho position was free for reaction with β-CD molecules. The spectrum of the MAA-β-CD was successfully depicted in Figure 2e. The major difference between MAA-TDI and MAA-β-CD spectra was the complete disappearance of the –NCO group at 2200 cm^−1^. This suggested that the unreacted ortho–NCO groups completely reacted with one of the primary –OH groups of β-CD subsequently forming urethane linkages. In addition, phenyl groups were recorded at the peaks at 1600, 1530, and 760 cm^−1^, while the O–C–O groups were recorded at the peaks at 1720, 1220, and 1070 cm^−1^. The spectrum showed consistency for the features of MAA and β-CD, and proved that the synthesis of MAA-β-CD was successfully performed.

**Figure 3:**
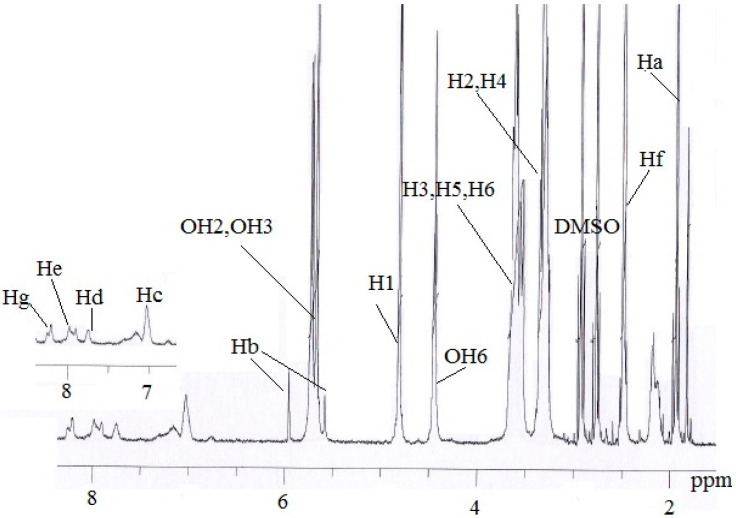
^1^H NMR spectrum of MAA-β-CD.

**Figure 4:**
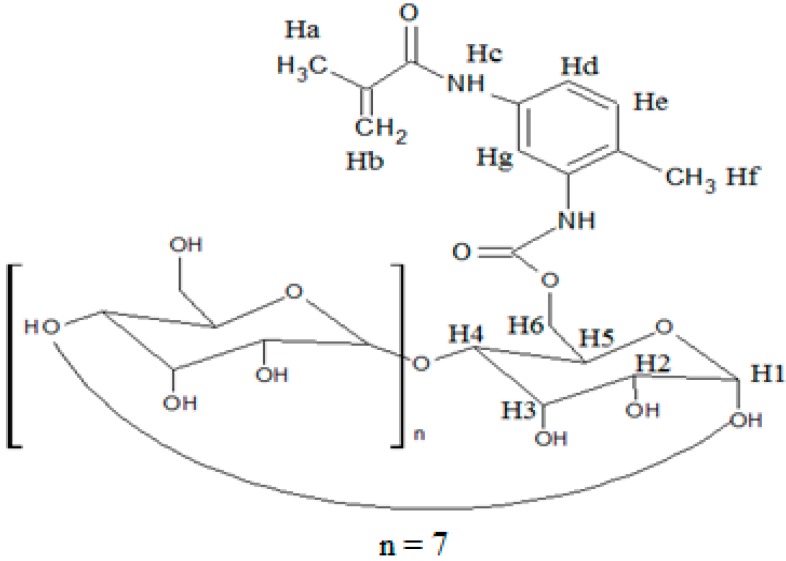
Formation of MAA-β-CD molecule structure.

The ^1^H NMR/ppm spectrum of the MAA-β-CD (Figure 3) was recorded in DMSO-d_6_ solvent. The result revealed a peak at 1.9750 (Ha) assigned for proton of CH_3_, and a peak at 5.7356 and 5.806 (Hb) assigned for protons of C=C. The tolyl group was observed at peak 2.5205 (Hf) for protons of CH_3_ and peaks at 7.7248 (Hc), 7.9205 (Hd), 8.2204 (He), and 8.4229 (Hg) for protons of the benzene ring. The peaks at 5.7356, 5.7179, 4.8118, 4.4487, 3.3272, 3.6213, 3.3620, 3.6012, and 3.6482 were, respectively, assigned to OH2, OH3, H1, OH6, H2, H3, H4, H5, and H6, corresponding to the functionalized β-CD molecule structure. From these results, the successful formation of MAA-β-CD was confirmed, as shown in Figure 4.

### 2.2. Characterization of MIPs/NIPs

#### 2.2.1. Fourier Transform Infrared (FTIR) Analysis

**Figure 5:**
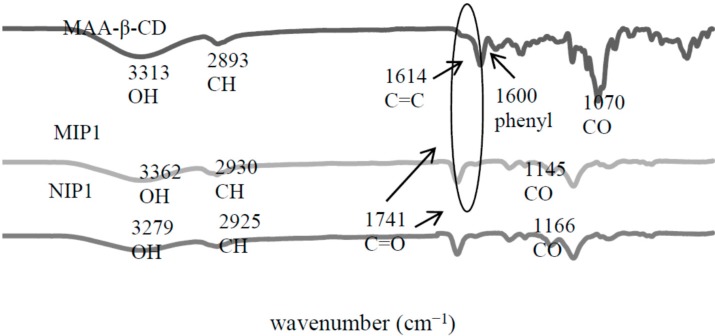
FTIR spectra of molecularly imprinted polymer-methacrylic acid functionalized β-cyclodextrin (MIP1) and non-molecularly imprinted polymer-methacrylic acid functionalized β-cyclodextrin (NIP1).

The imprinted polymers were characterized using FTIR by comparing them with their respective monomers. As shown in Figure 5, it was recorded that that MIP1 and NIP1 respective to the peaks at 3362 and 3371 cm^−1^, corresponded to the formation of the carbamate (–NHCO) group between –OH (β-CD) and N=C=O (MAA-TDI) from the monomer. The C–H group was located at peaks 2930 cm^−1^ (MIP1) and 2925 cm^−1^ (NIP1). The C–O group was assigned at peaks 1145 cm^−1^ (MIP1) and 1166 cm^−1^ (NIP1), while the C=O group was located at the peak 1741 cm^−1^. The FTIR spectra of MIP1 and NIP1 were rather similar to the MAA-β-CD spectrum. However, it could be seen that the polymerization was confirmed by the disappearance of the C=C at peak 1614 cm^−1^. It proved that C=C of MAA-β-CD and TRIM cross-linker had reacted with each other. This result successfully confirmed the polymerization of the MIP1/NIP1.

**Figure 6:**
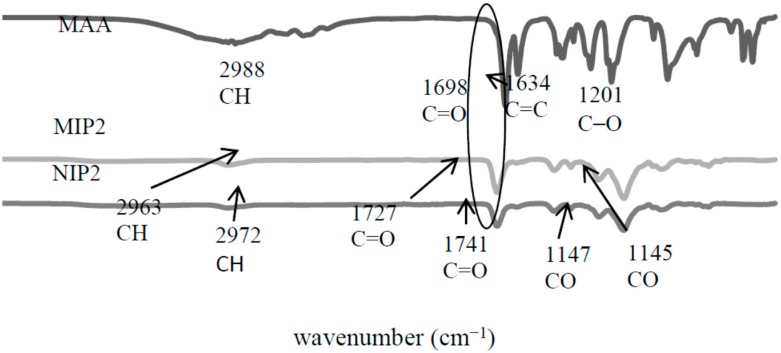
FTIR spectra of molecularly imprinted polymer-methacrylic acid (MIP2) and non-molecularly imprinted polymer-methacrylic acid (NIP2).

The FTIR spectrum of MAA in Figure 6 showed that the peak at 2988 cm^−1^ was corresponding to the C–H stretching vibration in MAA. The peak at 1698 cm^−1^ was assigned to the C=O group, and the peaks at 1634 and 1201 cm^−1^, respectively, assigned to the C=C and C–O groups. The spectra of MIP2 and NIP2 remained for several groups, such as C–H group, located at peak 2963 cm^−1^ (MIP2) and 2972 cm^−1^ (NIP2), while C=O was assigned at peak 1727 cm^−1^ (MIP2) and 1741 cm^−1^ (NIP2), and C–O was corresponded at peak 1145 cm^−1^ (MIP2) and 1147 cm^−1^ (NIP2). Similar observations like MIP1/NIP1 would be detected on the MIP2/NIP2 polymerization process, where the MIP2/NIP2 successfully polymerized due to the disappearance of C=C at peak 1698 cm^−1^. Generally, the absence of this peak strongly indicated the polymerization between the cross-linkers and monomers. These IR results successfully confirmed the formation of the MIPs/NIPs polymerization.

#### 2.2.2. Field Emission Scanning Electron Microscopy (FESEM) Analysis

**Figure 7:**
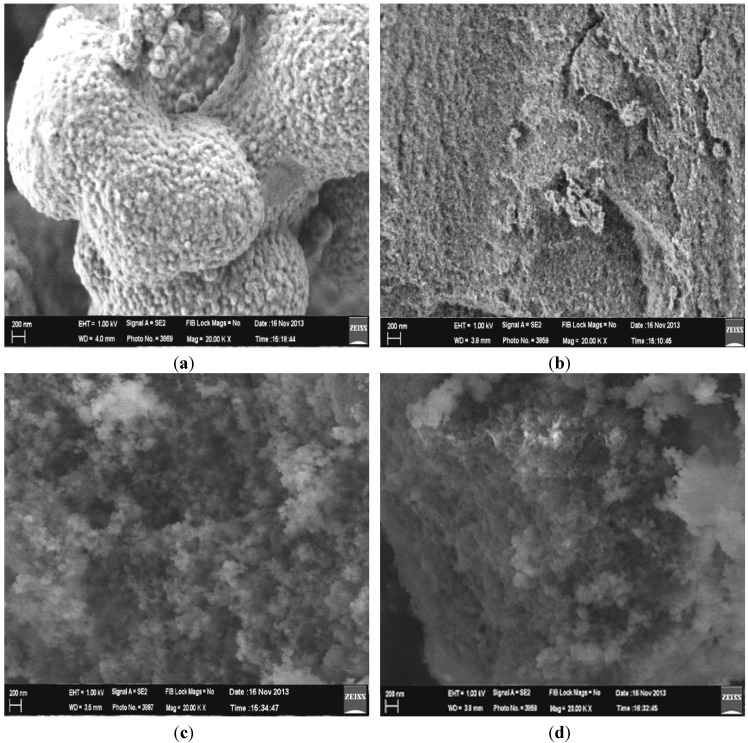
FESEM micrographs of (**a**) MIP1; (**b**) NIP1; (**c**) MIP2; (**d**) NIP2.

The microscopic morphological structures of MIPs/NIPs were identified using FESEM under 20,000× magnification. As shown in Figure 7, significant differences between MIP1/NIP1 and MIP2/NIP2 can be observed from the morphological images. It can be seen that MIP1 exhibited a spherical and spongy-porous morphology, while NIP1 showed a little roughness and cracked surface. It is suggested that the template factor affected the morphological structure of MIP1. The particles of MIP1 were spherical with a lot of porosity between them, which may be caused by the extracted BzP that was originally embedded in the MIP1 when the polymer was being prepared. However, the large pore size can be seen in the case of MIP1 compared with NIP1. The NIP1 appeared to have similar roughness, but it lacks the globules seen for MIP1. Since β-CD has hydrophilic characteristic, this might be attributed to the adsorption of water [27], which interrupts the polymer network. This impact might increase the globular appearance of MIP1, and more compact structure of NIP1 was formed, after water was removed.

Nevertheless, MIP2 and NIP2 exhibited uniform morphology, and did not show any detectable differences in both morphologies. Sometimes, the cavities caused from the molecular templates could not be observed from the morphological image analyses [9,28]. Several reports identified that the type of the crosslinking monomer, initiator, temperature, time, and stirring speed during the polymerization process could influence the formation of the morphology of MIP/NIP [8,29]. In this study, the possibility of monomers and solvents [30] strongly influences the modification morphology of the MIPs/NIPs polymer. The different morphologies between MIP1/NIP1 with MIP2/NIP2 confirmed the successful attachments of β-CD in the polymer matrix of MIP1/NIP1. It is revealed that the moiety of β-CD, which was fully accommodated inside the entire polymer matrix, strongly affected the particle growth and morphological design [30,31,32,33]. In addition, the utilization of DMAC and toluene solvents for MIP1/NIP1 and MIP2/NIP2 polymerization, respectively, could also alter their morphological structure.

#### 2.2.3. Particle Size and Brunauer-Emmett-Teller (BET) Analysis

The BET data (Table 1) showed that all *S*, *d_p_*, and *V_p_* values of MIPs were higher than their controls (NIPs). This was caused by the presence of the cavity recognition sites of the template in the MIPs polymer matrix, hence confirming the possibility of higher binding capacity of MIPs than the NIPs [32]. The BET values of MIP2 (426.9 m^2^/g) and NIP2 (415.0 m^2^/g) agreed with Spivak (2005) [30], which described that the typical values for the surface area of the imprinted polymer are in the range of 100–400 m^2^/g, while the average pore diameter in mesoporous form is in the range of 2–100 nm in diameter. The MIP1/NIP1 polymers were successfully confirmed, when the addition of the moiety of β-CD into the imprinted polymer matrix decreased the surface area values in the dry state, which were observed to be 11.31 m^2^/g (MIP1) and 2.22 m^2^/g (NIP1) compared to MIP2/NIP2, while the pore diameter values for MIP1/NIP1 (8.67/8.21 nm) were confirmed to be mesoporous, which were slightly higher than those of MIP2/NIP2 (7.14/6.01 nm). The total pore volume values of the MIP1/NIP1 were lower (4.20 × 10^−4^/5.75 × 10^−4^ cm^3^/g) than those of MIP2/NIP2 (0.7/0.6 cm^3^/g). This proved the hydrogel nature of MIP1/NIP1, complete with its high swelling capacity in water and with many cavities, making its network expandable to allow for a rapid diffusion process for the adsorbed analyte. This basically means that the surface area, pore diameter, and pore volume of MIP1/NIP1 could theoretically increase after swelling [34]. It might also serve as evidence of the fact that β-CD was mainly isolated inside the internal surfaces of the polymers [35]. It was proven that the isolated β-CD was not the main component being cross-linked with the TRIM cross-linker, but MAA located on the surface of the polymer matrix that interacted with the TRIM cross-linker. In short, the utilization of a different monomers and solvents does not only influence the morphological structure, but they also potentially plays a role in the particle growth of the imprinting polymer, and act as effective variables in controlling specific surface area, total pore diameter, and pore volume distributions [30].

**Table 1:** Brunauer-Emmett-Teller (BET) results.

Samples	Surface Area, S (m^2^/g)	Pore Diameter, dp (nm)	Pore Volume, Vp (cm^3^/g)
MIP1	11.31	8.67	4.20 × 10^−4^
NIP1	2.22	8.21	5.75 × 10^−4^
MIP2	426.9	7.14	0.7
NIP2	415.0	6.01	0.6

**Figure 8:**
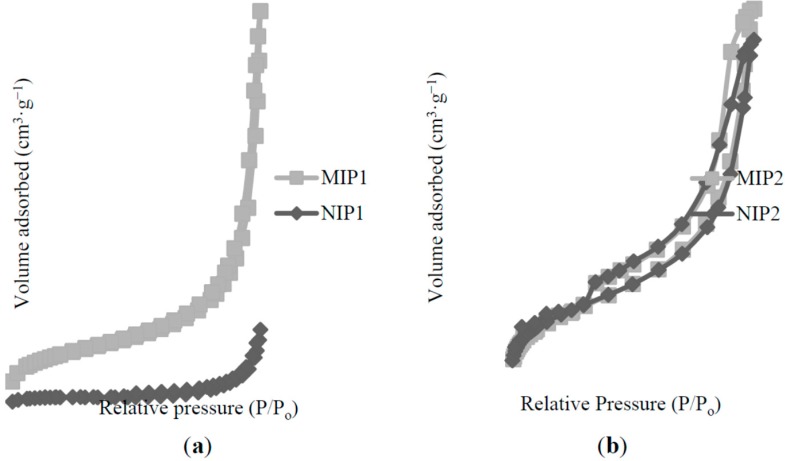
Nitrogen adsorption/desorption profiles of (**a**) MIP1/NIP1; (**b**) MIP2/NIP2.

The corresponding origin N_2_ adsorption isotherms for all of the polymers were presented in Figure 8. The shape of their N_2_ adsorption/desorption isotherm was a type-IV, suggesting that all polymers possess a mesoporous structure, leading to a hysteresis loop [36]. A high volume adsorbed of MIP2/NIP2 resulted in a large surface area compared to MIP1/NIP1, which was agreed in Table 1. A desorption branch of MIP1/NIP1 and MIP2/NIP2 exhibited a clear hysteresis loop between *P*/*P*_0_ = 0.90 and 0.40, indicative of the formation of “bottleneck” type of pores [31]. However, the MIP1/NIP1 figures showed a sharp elevation in the adsorbed *N*_2_ volume for *P*/*P*_0_ > 0.9. This feature was attributable to the presence of high textural (inter-particle) porosity [31], and such particle domains represent the substantial adsorption of nitrogen for low surface area mesoporous materials [37]. A rapid increase in the volume of adsorbed *N*_2_ for MIP2/NIP2, observed at a range of *P*/*P*_0_ 0.4–0.9, may be attributed to the delay condensation, indicating the excellent homogeneity of polymers and fairly small pore sizes [38].

Both MIP1/NIP1 and MIP2/NIP2 exhibited a Type H3 (adsorption) hysteresis loop, which did not exhibit any limiting adsorption at high *P*/*P*_0_, indicating the fact that the polymers consisted of aggregates of plates, giving rise to the slit-shape mesoporous structures [39]. However, slightly different hysteresis loops were spotted between MIP1/NIP1 and MIP2/NIP2 for a desorption branch of hysteresis loops. The MIP1/NIP1 exhibited Type H1 (desorption), proving the fact that the polymer has agglomerates or compacts of spheroidal particles that are fairly uniform in size and shape [40]. The desorption patterns of both MIP2/NIP2 would be regarded as Type H4, which is often associated with narrow slit-like pores [36]. Type H4 hysteresis contain a characteristic step-down in the desorption branch that are associated with the hysteresis loops’ closure [41].

### 2.3. Binding Characteristics of the Polymers for BzP

Binding experiments were carried out to investigate the affinity of the imprinted polymer for BzP. The binding affinities of the polymers were evaluated by the distribution coefficients of BzP between the polymer and the solution. The distribution coefficients (*K_d_*) is defined as [14]:

*K_d_* (L/g) = *C_p_/C_f_* (1)

where *C_p_* is the amount of BzP bound per gram of supports, and it was calculated according to the equation:

*C_p_* (mg/g) = *Q* (mg)/mass of polymer (g) (2)

The molecular imprinting factor (*IF*) was used to evaluate the imprinting effect. *IF* was calculated according to the equation:

*IF* = *K_d_* (MIP)/*K_d_* (NIP) (3)

The obtained distribution coefficients, *K_d_* and the *IF* values, were listed in Table 2. The *K_d_* and *IF* values of MIP1 were higher than MIP2. An *IF* value, higher than 1.5 was considered an imprinted polymer with an excellent selective recognition for the adsorbate [42]. It was suggested that the MIP1 had higher binding affinity compared to MIP2, towards BzP. It was attributed to the collaboration of triple interaction mechanism, including inclusion complex, hydrogen bonding, and π–π interaction (will described in Section 3.4). The mechanisms were crucial for the efficient improvement in the affinity and specificity for the guest of the imprinted polymer. It was proven that the β-CD molecule improved the recognition accuracy and selective binding of the MIP1. The MIP2 has a low binding affinity than MIP1, indicating only the presence of hydrogen bonding between BzP and MAA monomer.

**Table 2:** Recognition of BzP on the MIPs and NIPs.

Polymers	*K_d_* (L/g)	*IF*
MIP1	1.901 ± 0.025	7.979
NIP1	0.238 ± 0.044	-
MIP2	1.518 ± 0.071	1.733
NIP2	0.876 ± 0.054	-

In order to verify the selectivity of MIP1 and MIP2 for BzP, the selectivity test was performed using certain structurally related substrates. A similar structural analogue of parabens, which included butylparaben (BuP), propylparaben (PrP), ethylparaben (EtP), and methylparaben (MeP), were compared with BzP. The obtained values of *K_d_* and *IF* for the substrates were shown in Table 3. MIP1 and MIP2 adsorbed the other parabens, suggesting the presence of certain cross-binding reactivity [43]. Nevertheless, the *K_d_* values showed that MIP1 and MIP2 exhibited high-binding specificity for BzP compared to all other tested parabens. The presence of certain cross-binding reactivity in the MIPs could be an advantage in sample treatments, because a class of template analogues could also be removed or enriched in an efficient manner. Compared with MIP2, MIP1 is more suitable as an adsorbent for this application in the context of imprinting factor. Furthermore, NIP1 and NIP2 exhibited low values of *K_d_* for all parabens, with no imprinting effect for MeP. This evidence indicated that the imprinting method created a microenvironment based on the shape selection and position of functional groups that recognized the BzP template molecule. This result also illuminated aspects of the molecular recognition mechanism.

**Table 3:** *K_d_* and *IF* values of the parabens on polymers. “-” means the test was done but the datum could not be calculated; results based on three replicate analyses for all analytes.

Parabens	MIP1	NIP1	*IF*	MIP2	NIP2	*IF*
	*K_d_* (L/g)	*K_d_* (L/g)		*K_d_* (L/g)	*K_d_* (L/g)	
**Benzylparaben (BzP)**	1.901 ± 0.025	0.238 ± 0.044	7.979	1.518 ± 0.071	0.876 ± 0.054	1.733
**Butylparaben (BuP)**	0.675 ± 0.031	0.113 ± 0.018	5.991	0.787 ± 0.097	0.477 ± 0.002	1.649
**Propylparaben (PrP)**	1.048 ± 0.063	0.189 ± 0.014	5.538	0.419 ± 0.035	0.239 ± 0.012	1.751
**Ethylparaben (EtP)**	0.389 ± 0.002	0.049 ± 0.004	7.832	0.210 ± 0.040	0.201 ± 0.049	1.044
**Methylparaben (MeP)**	0.171 ± 0.007	-	-	0.059 ± 0.003	-	-

**Figure 9:**
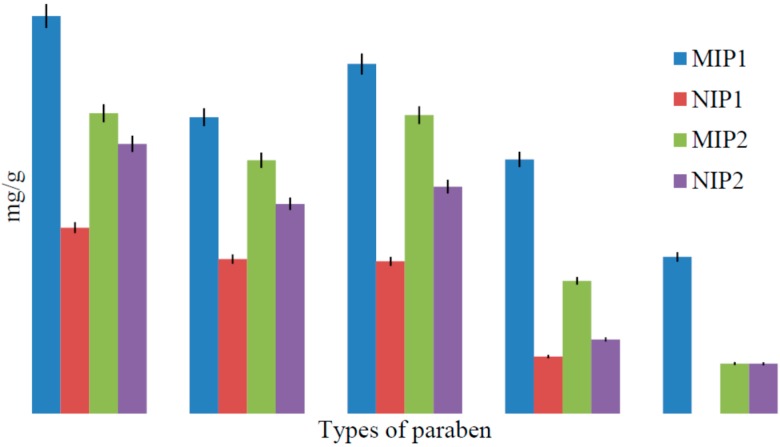
Binding specificity of BzP on MIP1/NIP1 and MIP2/NIP2.

Figure 9 revealed that the MIP1 had a greater adsorption capacity than NIP1, corresponding to the *K_d_* values in Table 3. It is clearly determined that the high specific recognition sites were left by the BzP template on the MIP1, but were non-specific in NIP1. Moreover, the MIP1 had a higher binding capacity than MIP2, which corresponded to *K_d_* and *IF* values, indicating that the β-CD molecule contributed to the improvement of the recognition accuracy and selective binding of the MIP1, identifying that MIP1 had excellent performance behavior.

### 2.4. Inclusion Complex of MAA-β-CD-BzP

In order to get information of the binding specificity mechanism between MAA-β-CD monomer with BzP template, the analysis of the inclusion complex between MAA-β-CD and BzP was crucial, since the cavity of β-CD was maintained during the MIP polymerization process. Due to the hydrophobic nature of BzP template and hydrophobic core of β-CD, the BzP template should be inserted into the cavity of the β-CD residues. The DSC result in the experimental section confirmed the formation of MAA-β-CD-BzP inclusion complex. Continuously, the ^1^H NMR analysis was conducted to investigate the formation of the inclusion complex between MAA-β-CD and BzP. The chemical shift changes of the specific nuclei in the host molecule (MAA-β-CD) can verify the formation of the inclusion complex in the solution, since significant changes in the microenvironment are known to occur in the CD of the inclusion complex [44,45,46]. Table 4 illustrated the confirmation of the MAA-β-CD-BzP inclusion complex.

**Figure 10:**
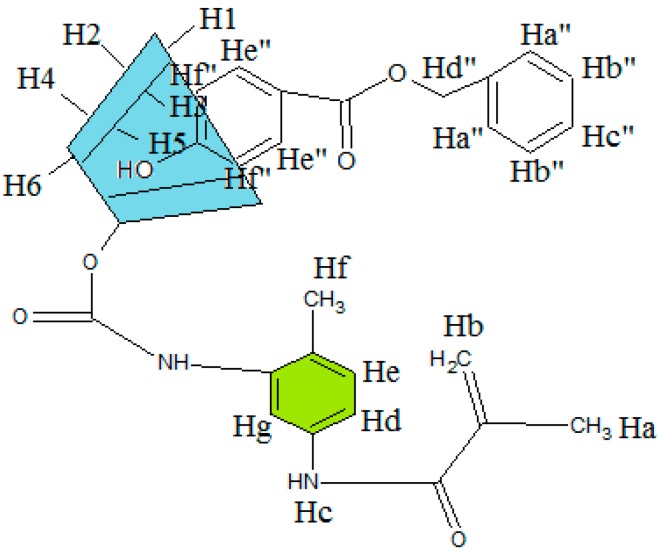
Proposed structure of possible inclusion complex of methacrylic acid functionalized β-cyclodextrin with benzylparaben (MAA-β-CD-BzP) mechanism.

Table 4 observed that the chemical shifts of protons H3 and H5 (interior cavity of β-CD) were slightly higher compared to other protons, such as H1, H2, H4 and H6 (exterior cavity β-CD). This clearly proved that during the inclusion complex formation, the screening environment could only be sensed for the hydrogens on the internal surface (H-3 and H-5) and not by the hydrogens on the external surface [47]. Meanwhile, the protons Hf'' and OH of the BzP indicated higher values compared to other protons. This led to the assumption that the phenyl ring of the BzP entered the hydrophobic cavity of the β-CD. The proposed structure of inclusion complex between MAA-β-CD and BzP is presented in Figure 10. The result revealed that the environments of these protons were altered after the inclusion.

**Table 4:** ^1^H NMR chemical shift (ppm) for MAA-β-CD, BzP, and MAA-β-CD-BzP.

H Proton	δ (MAA-β-CD-BzP)	δ (MAA-β-CD)	δ (BzP)	Δδ
Ha	1.9750	1.9750	-	0.0000
Hb	5.6673	5.7356	-	−0.0683
Hc	7.7481	7.7248	-	0.0233
Hd	7.9079	7.9205	-	−0.0126
He	8.2088	8.2204	-	−0.0116
Hf	2.5205	2.5205	-	0.0000
Hg	8.4547	8.4229	-	0.0318
H1	4.8124	4.8118	-	0.0006
H2	3.2900	3.3272	-	−0.0372
H3	3.5365	3.6213	-	−0.0848
H4	3.3205	3.3620	-	−0.0415
H5	3.3510	3.6012	-	−0.2502
H6	3.6213	3.6482	-	−0.0269
OH2	5.7332	5.7356	-	−0.0024
OH3	5.7155	5.7179	-	−0.0024
OH6	4.4469	4.4487	-	−0.0018
Hc''	7.3856	-	7.3844	0.0012
Hd''	5.2762	-	5.2786	−0.0024
He''	7.8463	-	7.8493	−0.0030
Hf''	6.8517	-	6.8559	−0.0042
OH	10.3426	-	10.3481	−0.0055

**Figure 11:**
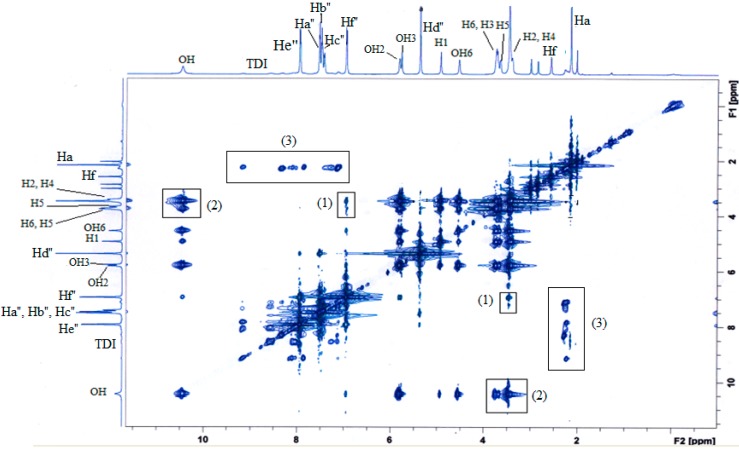
2D NOESY spectrum of MAA-β-CD-BzP inclusion complex; (1) cross peaks of protons Hf'' of BzP with H3 and H5 of β-CD; (2) cross peaks of protons OH of BzP with H3 and H5 of β-CD; (3) cross peaks of protons Ha of MAA and BzP, respectively.

However, since there are two rings (benzene and phenyl ring) present in the BzP structure, two-dimensional nuclear Overhauser effect spectroscopy (2D NOESY) was adopted to determine which aromatic ring of the BzP were encapsulated by the MAA-β-CD’s cavity, and it successfully predicted that it was the guest molecule that was inside the β-CD cavity. Figure 11 illustrated the 2D NOESY spectral data by ^1^H–^1^H cross connection peaks between the MAA-β-CD and BzP. The 2D NOESY spectral data showed that the proton of Hf'' of BzP was clearly located in the space cavity of protons H3 and H5 for β-CD (shown in box (1)). Meanwhile, the 2D NOESY spectral data clearly indicated that OH for BzP obviously entering the space cavity of protons H3 and H5 for β-CD (shown in box (2)). The NOESY spectra strongly suggested that the phenyl ring of BzP was encapsulated inside the cavity of β-CD via an inclusion complex. Additionally, the strong intensity could be observed at the cross-peaks of protons Ha for MAA with the BzP protons (shown in box (3)). Overall, the strong correlation revealed that the BzP template was strongly interacting with the MAA-β-CD monomer.

**Scheme 1:**
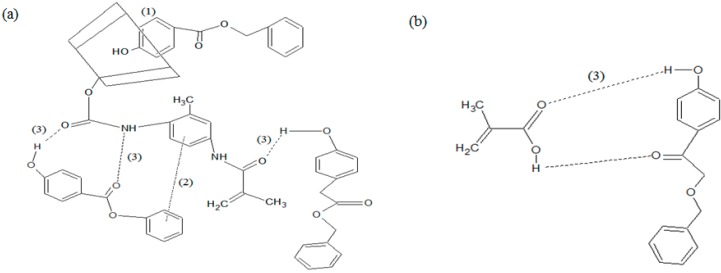
Interaction mechanism between monomer (**a**) MAA-β-CD; (**b**) MAA with BzP; where (1) inclusion complex; (2) π–π interaction; and (3) hydrogen bonding.

The interaction of MAA-β-CD with BzP in the MIP1 adsorption system could have possibly formed in Scheme 1, and has been proposed by taking into account the hydrogen bonding between the amido groups of MAA-β-CD with the ester group of BzP. The inclusion complex possibly occurred between the β-CD with the phenyl ring of the BzP. The π–π interaction took place between aromatic ring of BzP (π donor) and the aromatic ring of isocyanate ring of MAA-β-CD (π acceptor). However, the interaction between MIP2 and BzP is based only on hydrogen bonding. Poor specific binding and low binding adsorption for MIP2 could possibly be explained by the sole hydrogen bonding between both hydroxyl and carbonyl groups of MAA and BzP structures. It was proven that the modification of monomer based on β-CD provided the possibility of better binding capacity of MIP1 as opposed to MIP2.

## 3. Experimental Section

### 3.1. Materials and Methods

Benzylparaben (BzP), butylparaben (BuP), propylparaben (PrP), ethylparaben (EtP), methylparaben (MeP), toluene-2,4-diisocyanate (TDI), methacrylic acid (MAA), trimethylolpropane trimethacrylate (TRIM), benzyol peroxide (BPO), dimethylacetamide (DMAC), methanol (MeOH), acetic acid, toluene, 2,2-phenyl-2-propyl benzodithioate (PPDT), and dibutyl dilaurate (DBTDL) were purchased from Sigma-Aldrich (Buches SG, Switzerland). β-cyclodextrin (β-CD) (99%) was commercially available, and was purchased from Acros (Geel, Belgium). Other reagents and chemicals were of analytical reagent grade, and were used as received without further purification. Distilled water was used throughout the experiments.

### 3.2. Instruments

The FTIR spectra of the polymers were recorded using the FTIR spectrophotometer (Perkin Elmer, Perkin-Elmer Waltham, MA, USA) in KBr pellets. The analysis of the BET surface area and porous properties of the polymers were determined from the nitrogen adsorption-desorption analysis at 77 K using a surface area analyzer (Quantachrome, Boynton Beach, FL, USA). The specific surface area, average pore diameter, and total pore volume were calculated from Brunauer-Emmett-Teller (BET), Barrett-Joyner-Halenda (BJH) method, and Dubinin–Radushkevich (DR) plots, respectively. The morphologies of the polymers were shown by Quanta FEG 450 Field Emission Scanning Electron Microscope (FESEM) (FEI, Hillsboro, OR, USA). Differential Scanning Calorimeter (DSC) (Perkin Elmer, Waltham, MA, USA) analysis was done by heating the samples from 30 to 400 °C at 20 °C per minute. The Proton Nuclear Magnetic Resonance (^1^H NMR) and two-dimensional Nuclear Overhauser effect Spectroscopy (2D NOESY) spectra of the inclusion complex samples in dimethyl sulfoxide (DMSO) were recorded on a Lambda JEOL 400 MHz Fourier Transform NMR (FT-NMR) spectrometer (Bruker, Fӓllanden, Switzerland) at room temperature. The rebinding experiments were conducted using a Shimadzu Ultraviolet-Visible spectroscopy (UV-Vis) recording spectrophotometer (Shimadzu, Tokyo, Japan), equipped with 1 cm quartz cells.

### 3.3. Synthesis of MAA-β-CD Monomer

**Scheme 2:**
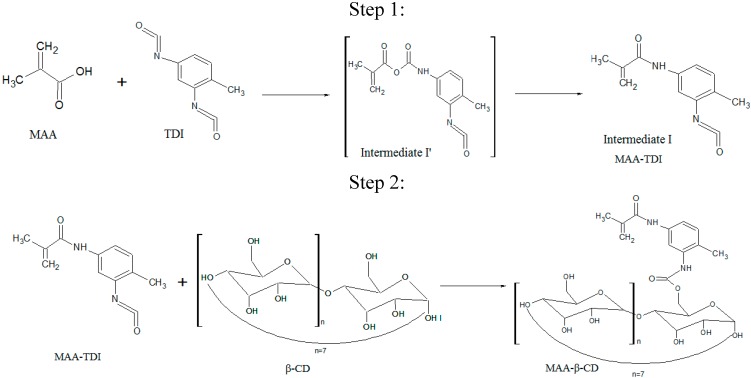
Synthesis of MAA-β-CD.

The method was adopted from Sreenivasan (1996) [7]. The molar concentration was chosen in this case, using a stoichiometry ratio of 0.5 M MAA: 1 M TDI: 0.5 M β-CD. An amount of 2.855 mL of TDI with 0.848 mL of MAA was mixed in 20 mL of DMAC solvent. Then, 0.1% (0.02 mL) of DBTDL catalyst was added. The solution was magnetically stirred at room temperature under nitrogen gas for an hour. The solution at this stage was subjected to the FTIR analysis represented as MAA-TDI (Figure 3c). Then, 0.5 M (14.19 g) of β-CD was poured to the mixture, and a further 5 mL of DMAC was added. The mixture was again stirred for 2 h, and the resultant MAA-β-CD was produced. The synthesis route of MAA-β-CD was represented in Scheme 2. It showed that the reaction between MAA and TDI was stoichiometric. It can be seen in step 1, that the intermediate I' containing an anhydride and carbamate group was formed at the consumption of carboxyl group in MAA. Intermediate I’ is unstable [48], and it converts to methacrylic amide containing an isocyanate group. Intermediate I contains methacrylic amide and –NCO, indicating that only one of the two –NCO groups in TDI took part in the reaction. The unreacted –NCO in I can be used for further isocyanation with β-CD to product MAA-β-CD, as shown in step 2. The formation of MAA-β-CD was confirmed using FTIR and ^1^HNMR spectroscopies.

### 3.4. Synthesis of MAA-β-CD-BzP Complex

**Figure 12:**
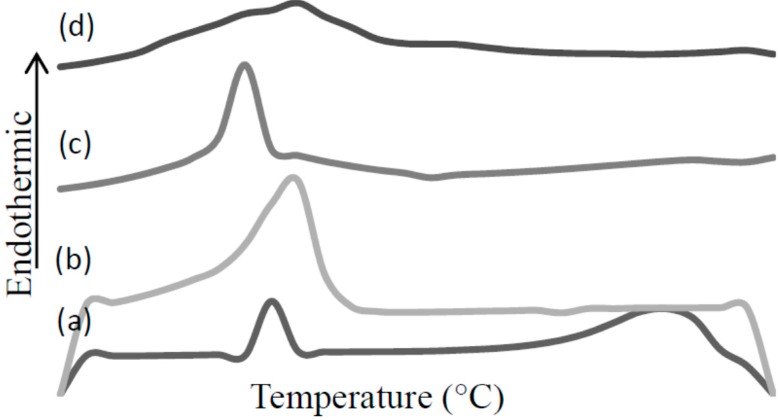
Differential Scanning Calorimeter (DSC) curves of (a) BzP; (b) MAA-β-CD; (c) MAA-β-CD-BzP physical mixture; (d) MAA-β-CD-BzP complex.

The complex of MAA-β-CD with BzP was synthesized using the conventional kneading method [45]. Equimolar amounts of MAA-β-CD and BzP were kneaded with mortar and pestle in the minimal presence of ethanol to form a homogeneous paste. The complex was kneaded for approximately 30 min, and dried to a constant mass. After drying, a pale yellow powder (MAA-β-CD-BzP complex) was obtained. In the present work, DSC was used to analyze BzP, MAA-β-CD, MAA-β-CD-BzP physical mixture, and MAA-β-CD-BzP complex. As shown in Figure 12, DSC thermograms revealed marked structural differences between the physical mixture and the complex. The thermal profile of the BzP, MAA-β-CD, and the physical mixture showed the endothermic peaks at 110, 130, and 100 °C, respectively. In a thermal physical mixture, the single appearance peak indicated that both BzP and MAA-β-CD were thoroughly mixed, but were able to maintain their original crystalline structures in the mixture. However, a different pattern was observed in the thermogram of the complex, as a new broad peak appeared at 120 °C. These results were indicative of the changes in the structure of the substrates and the tight interaction between BzP and MAA-β-CD.

### 3.5. Synthesis of MIPs/NIPs

**Scheme 3:**
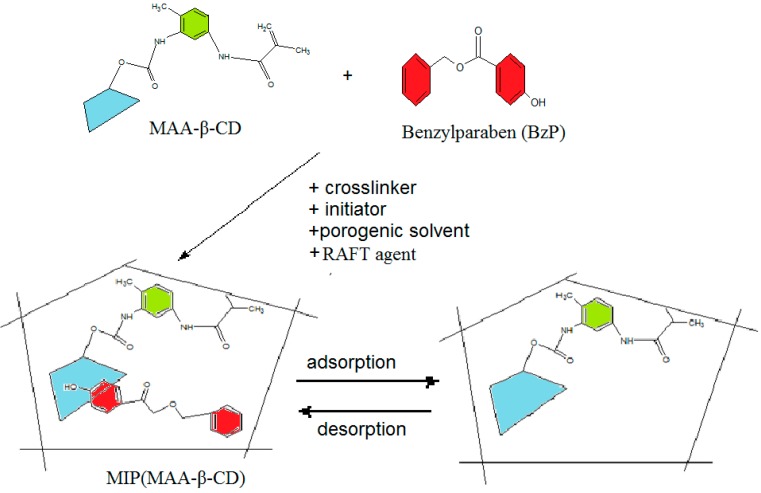
Preparation of molecularly imprinted polymer-methacrylic acid functionalized β-cyclodextrin, MIP(MAA-β-CD).

For the synthesis of the MIP (MAA-β-CD) as MIP1 (Scheme 3), BzP template (0.14 mmoL, 0.032 g) was dissolved in 10 mL of DMAC solvent containing MAA-β-CD as monomer (0.56 mmoL, 0.757 g), TRIM as cross-linker (2.80 mmoL, 0.894 mL), 0.15 g of BPO as initiator, and PPDT as a RAFT agent (1.24 mmoL, 0.327 g). MIP (MAA) as MIP2, was synthesized with methacrylic acid (MAA) as a monomer, where β-CD was omitted. The pre-polymerization mixture comprised of BzP (0.14 mmoL, 0.032 g), MAA (0.56 mmoL, 0.049 mL), TRIM (2.80 mmoL, 0.8939 mL), BPO (0.15 g), and PPDT (1.24 mmoL, 0.327 g), and was dissolved in 10 mL of toluene (difference solvent was applied depending on the solubility of the monomer used). Then, the solution was sealed and purged with nitrogen gas for at least 10 min before placing it in a water bath at 70 °C overnight. After polymerization, the obtained MIPs were crushed, ground, and wet-sieved. The extraction process was carried out with a mixture of methanol/acetic acid (*v*/*v*, 9:1) until the BzP in the elution could no longer be detected at 258 nm by the UV-Vis spectrophotometer. Then, the MIPs particles were washed with methanol to remove any residual acetic acid and dried under vacuum at 80 °C. Similar procedures were carried out for non-molecularly imprinted polymers, which were non-molecularly imprinted polymer-methacrylic acid functionalized β-cyclodextrin, NIP(MAA-β-CD) and non-molecularly imprinted polymer-methacrylic acid, NIP(MAA), represented as NIP1 and NIP2, without the template, as references.

### 3.6. Binding Experiments

The MIPs/NIPs polymer particles (0.05 g) were placed in a 20 mL centrifuge tube and mixed with 10 mg/L of the selected substrate solution, diluted with ethanol/water (1/10, *v*/*v*) mixture. The mixture was shaken for an hour at room temperature. Then, the mixture was filtered, and the concentration of the substrate solution was determined by the spectrophotometer. The amount of substrate bound to the polymer (*Q*) was calculated according to equation:

*Q* = *V* (*C_i_* − *C_f_*) (4)

where *C_i_* and *C_f_* represent the initial and final concentrations (mg/L), and *V* is the volume (L) of solution, respectively.

## 4. Conclusions

MIP(MAA-β-CD), which was synthesized with MAA-β-CD monomer was successfully examined. The morphological structure of the MIP(MAA-β-CD) exhibited a sponge-porous structure. BET data proved that the presence of β-CD in MIP(MAA-β-CD) strongly affected the particle growth of MIP polymer during the polymerization process. The relative information on the binding specificity mechanism of MIP(MAA-β-CD) was obtained from DSC analysis, and supported by ^1^H NMR and 2D NOESY experiments. The study revealed that the collaboration of triple interactions (hydrogen bonding, π–π interaction, and inclusion complex) between the monomer and the template was essential in enhancing the affinity and specificity of the imprinted polymer. Again, β-CD significantly contributed to the enhancement of the morphology, recognition affinity, and selective adsorption of the MIP performance.
